# Towards a universal influenza vaccine: different approaches for one goal

**DOI:** 10.1186/s12985-017-0918-y

**Published:** 2018-01-19

**Authors:** Giuseppe A. Sautto, Greg A. Kirchenbaum, Ted M. Ross

**Affiliations:** 1 Center for Vaccines and Immunology, University of Georgia, Athens, GA USA; 2 Department of Infectious Diseases, University of Georgia, Athens, GA USA

**Keywords:** Influenza virus, Vaccine, Hemagglutinin (HA), HA head, HA stem, Monoclonal antibodies (mAbs)

## Abstract

Influenza virus infection is an ongoing health and economic burden causing epidemics with pandemic potential, affecting 5–30% of the global population annually, and is responsible for millions of hospitalizations and thousands of deaths each year. Annual influenza vaccination is the primary prophylactic countermeasure aimed at limiting influenza burden. However, the effectiveness of current influenza vaccines are limited because they only confer protective immunity when there is antigenic similarity between the selected vaccine strains and circulating influenza isolates. The major targets of the antibody response against influenza virus are the surface glycoprotein antigens hemagglutinin (HA) and neuraminidase (NA). Hypervariability of the amino acid sequences encoding HA and NA is largely responsible for epidemic and pandemic influenza outbreaks, and are the consequence of antigenic drift or shift, respectively. For this reason, if an antigenic mismatch exists between the current vaccine and circulating influenza isolates, vaccinated people may not be afforded complete protection. There is currently an unmet need to develop an effective “broadly-reactive” or “universal” influenza vaccine capable of conferring protection against both seasonal and newly emerging pre-pandemic strains. A number of novel influenza vaccine approaches are currently under evaluation. One approach is the elicitation of an immune response against the “Achille’s heel” of the virus, i.e. conserved viral proteins or protein regions shared amongst seasonal and pre-pandemic strains. Alternatively, other approaches aim toward eliciting a broader immune response capable of conferring protection against the diversity of currently circulating seasonal influenza strains.

In this review, the most promising under-development universal vaccine approaches are discussed with an emphasis on those targeting the HA glycoprotein. In particular, their strengths and potential short-comings are discussed. Ultimately, the upcoming clinical evaluation of these universal vaccine approaches will be fundamental to determine their effectiveness against preventing influenza virus infection and/or reducing transmission and disease severity.

## Background

The high degree of variability amongst influenza viruses is the main characteristic that provides the greatest challenge to development of prophylactic and therapeutic solutions against epidemic and pandemic outbreaks. In particular, current influenza vaccines do not confer complete protection against circulating epidemic and pandemic influenza strains.

New approaches are currently under investigation for development of more broadly-reactive or universal influenza vaccines. Several of these new approaches focus on the surface receptor-binding glycoprotein of the influenza virus, the hemagglutinin (HA), which is comprised of globular head and stem (or stalk) regions.

Given the conserved nature of the stem region, stem-based vaccine approaches aim to elicit antibodies that recognize both homosubtypic and heterosubtypic strains. However, the protective efficacy afforded by stem-directed antibodies is not completely clear.

The globular head of HA contains the receptor binding site and antibodies targeting this region inhibit virus binding to target cells. Approaches aimed at eliciting broad spectrum immune responses against the HA globular head are hindered by the high variability of epitopes in this region. Different strategies have been adopted to overcome this hurdle, including computationally optimized broadly reactive antigens (COBRA).

Overall, these novel head- and stem-based approaches are moving closer to a more broadly-reactive or universal influenza vaccine. However, there are additional aspects that deserve further considerations, such as the role of pre-existing immunity to influenza and how it shapes the response to vaccination, as well as age-related factors, that could influence the prophylactic effectiveness of current and candidate vaccines.

In this review we describe the current standard of care influenza vaccine, as well as those offering promise toward development of a universal influenza vaccination approach. In this context, vaccine approaches aimed at eliciting antibodies targeting the influenza HA glycoprotein are the primary focus.

## Introduction

### The influenza virus

Influenza viruses belong to the family *Orthomyxoviridae* and one of their major characteristic is the rapid rate of viral evolution. They are categorized into four types: A, B, C and D. Influenza virions, which have a diameter spanning from 80 to 120 nm, possess negative-sensed, single-stranded RNA genomes: 8 segments for influenza A and B or 7 for influenza C and D [1]. Influenza A viruses circulate in many avian species and infect several mammalian species including humans; influenza B viruses infect only humans; influenza C viruses infects humans and pigs and influenza D viruses primarily affect cattle and are not known to infect or cause illness in humans [2]. Influenza A viruses, together with influenza B viruses, are responsible for human seasonal epidemics and pre-pandemic outbreaks. Moreover, they cause respiratory illness in humans with the potential for severe complications in chronically compromised subjects. Annually, 3–5 million cases of serious illness caused by influenza virus infections resulting in 250,000 to 500,000 deaths worldwide can occur; however, pandemics have the potential to claim millions of human lives [3].

Each influenza A virus is further classified, on the basis of the surface glycoproteins hemagglutinin (HA) and neuraminidase (NA), into subtypes. At present, 18 HA and 11 NA subtypes have been identified circulating in birds and mammals. Notwithstanding the antigenic differences among the different HA proteins, there is a certain degree of antigenic relatedness that facilitates the clustering of influenza A viruses into two major phylogenetic groups: group 1 (which includes subtypes H1, H2, H5, H6, H8, H9, H11, H12, H13, H16, H17, and H18) and group 2 (which includes subtypes H3, H4, H7, H10, H14 and H15) [4–6]. Viruses with almost all combinations of HA and NA have been identified in avian species, while influenza viruses with a more restricted number of HA protein subtypes H1, H2, H3, H5, H6, H7 and H9 and H10 have been found in humans. In addition to influenza A viruses, two evolutionary diverging influenza B virus lineages have been reported: the Yamagata and the Victoria lineages [7].

Influenza A viruses responsible for seasonal influenza epidemics belong to the H1N1 and H3N2 subtypes and, together with influenza B viruses, are responsible for millions of infections each year. On the other hand, sporadic infection of humans with avian-origin influenza A viruses belonging to H2, H5, H6, H7, H9 and H10 HA subtypes can occur and poses the risk of instigating an influenza pandemic; however, these subtypes presently exhibit inefficient human-to-human transmissionability [8]. Furthermore, for the most hypervariable subtypes (such as H5N1), clades, subclades and even sub-subclades have been reported. Within a subtype, there is up to ~15% amino acid sequence diversity, whereas HA proteins between subtypes have 40% or 60% amino acid sequence diversity, which highlights the great hypervariable nature of these viral proteins [5].

Vaccination is one of the most effective means for public health control of infectious diseases such as influenza. In this review, we discuss the different approaches for influenza vaccination currently in use and experimental, novel promising strategies being tested, with a particular emphasis to those vaccines targeting the HA glycoprotein.

### The hemagglutinin (HA) glycoprotein of influenza virus

Hemagglutinin (HA) and neuraminidase (NA), are the main surface glycoproteins on influenza viral particles. NA is less abundantly expressed on the virion compared to HA (HA to NA ratio ranging from 4:1 to 5:1) and is responsible for cleavage of sialic acid moieties on the cell membrane to allow for release of nascent viral particles. Inhibition of NA enzymatic activity is the target of currently available anti-influenza drugs (oseltamivir), as well as anti-NA neutralizing antibodies [9].

The influenza HA is responsible for the binding to sialic acid, the receptor on target cells. There are approximately 500 molecules of HA per virion [10]. Owing to the pivotal role of HA in the viral life cycle, as well as its exposition on the viral envelope, this protein is the primary neutralizing target of the humoral immune response [11, 12]. HA is also an attractive molecule for the development of prophylactic and therapeutic approaches [13].

The mature form of the HA glycoprotein is a homotrimer of three HA monomers that are composed of a globular head and a stem region. The receptor binding site (RBS) resides in the globular head, which is also the most variable region of the protein, while the stem region is involved in the pH-induced fusion event triggered by endosome acidification following viral adsorption, and is more conserved amongst and across HA subtypes belonging to the same group [14].

The HA protein is synthesized as a single precursor, termed HA0, which is subsequently cleaved on the mature virion by cellular protease into two segments covalently linked to each other through a disulfide bond: HA1, which is the binding subunit and HA2, the fusion subunit. A stretch of hydrophobic amino acids in the N-terminus of the HA2 domain, which is particularly hidden in the HA structure, constitutes the so-called “fusion peptide” (FP) [14].

The host’s strong humoral immune response exerts pressure on HA that results in this antigenic diversity. HA is thus the most variable influenza protein and this antigenic diversity, mainly focused on the highly exposed HA1 subunit, is responsible for escape of influenza virus from pre-existing immunity [15]. The major mechanisms of HA and NA diversification can be attributed to antigenic drift and antigenic shift. Antigenic drift is characterized by the accumulation of mutations, especially at key antigenic sites in the HA globular head, due to the absence of the proofreading activity of the viral RNA polymerase and then to the selective pressure exerted by the host immune system [16]. In fact, the HA globular head contains the majority of the variable and immunodominant epitopes, whereas conserved and neutralizing epitopes within and outside the globular head regions have been strongly selected during evolution to be sub-dominant [12]. This phenomenon could be attributed to intrinsic structural features of these epitopes that make them immunologically silent or to their hindered nature which make them difficult to target [17].

Antigenic shift instead occurs through the introduction of a novel HA (and/or of a novel NA) subtype, derived from an animal reservoir (generally of avian or swine origin) and results in a gene reassortant event between these zoonotic influenza genomes and human influenza virus genomes. Introduction of an RNA segment encoding for an HA of novel subtype often results in an influenza virus with pandemic potential because the general population is immunologically naïve to this HA molecule [18]. The most well-known example of this phenomenon was the emergence of a novel H1N1 influenza virus in 1918–1919 causing the ‘Spanish flu’. More recent examples occurred in 1957, 1968 and 2009 [19].

## Currently available influenza vaccines

Current seasonal influenza vaccines are effective when the antigenicity of the strains used to generate the vaccines is closely matched with the respective circulating influenza A and B virus strains. However, these seasonal influenza vaccines need to be reformulated annually in order to elicit a protective antibody response that recognizes viral genetic variants that arise through antigenic drift. In detail, this process is conducted by the World Health Organization (WHO) Global Influenza Surveillance and Response System (GISRS) [20]. Of particular importance, these vaccines also do not confer protection against viruses with pre-pandemic potential causing outbreaks due to the emergence of viral strains with HA proteins from novel subtypes. Thus, a major thrust of new influenza vaccine research is to design immunogen(s) that not only protect(s) against current strains, but also future circulating strains resulting from antigenic drift and/or shift [21]. Currently, there are two formulations of the influenza vaccine: the inactivated influenza vaccine (IIV) and the live attenuated influenza vaccine (LAIV). Most of the commercially available influenza vaccines are manufactured by propagation of the virus in embryonated chicken eggs with a production time of 6–8 months, except the trivalent recombinant influenza vaccine expressed in baculovirus (FluBlok, by *Protein Sciences*) recently licensed by the FDA for human use and MDCK cell culture-based IIV (Flucelvax, by *Novartis*) [22, 23].

Therefore, considering the time factor, manufacturing large amounts of vaccine in short time period during an epidemic/pandemic is challenging. Moreover, the flu season is October–May in the Northern Hemisphere and May–October in the Southern Hemisphere. Furthermore, preparedness against influenza infection is compromised due to unpredictable variation of circulating strains compared to those annually selected to be included in the vaccine formulations. At present, commercially available influenza vaccines are either trivalent or quadrivalent formulations, which include an H1N1 strain, an H3N2 strain and 1 or 2 influenza B strains belonging to evolutionarily diverging lineages [24]. The efficacy of these vaccine formulations are highly variable amongst the global population, with an average of 50–60% estimated protection [25].

## Influenza vaccines under-development

### Stem-based approaches

Influenza virus infection elicits neutralizing antibodies against both the globular head and stem structures of the HA protein. There are vaccine strategies in development aimed at eliciting antibodies targeting the conserved stem region of HA [26]. In fact, given the higher immunodominance of head epitopes, current influenza vaccines minimally induce stem-directed humoral immunity [27].

Approaches to elicit stem-directed antibodies include sequential immunization with heterologous influenza strains, immunization with modified proteins by removing or glycan-masking the globular head, referred to as headless HA, through minimizing epitopes of the stem region, i.e. mini-stem proteins [28, 29], and hyperglycosylated HA head domain, respectively [30]. Each of these approaches are discussed in greater detail in the following sections.

### Sequential immunization and chimeric HA proteins

The concept of sequential immunization arose from the observation in humans that infection with the pandemic H1N1 virus elicited a boost in titer of antibodies directed against the hemagglutinin stem region [31]. Similarly, it has been confirmed in animal studies that infection/vaccination with the pandemic H1N1 virus followed by infection/vaccination with an antigen containing a novel head domain but the same stem region, elicited antibodies directed towards the stem region and less towards the globular head region [32–34]. In this regard, an immunization approach utilizing chimeric HA constructs which present novel globular head domains to the immune system in the context of a common stem backbone elicited an antibody response conferring heterosubtypic immunity [35]. Recently, Nachbagauer et al., described this approach in the context of a LAIV bearing an H8 head domain and an H1 stem domain (cH8/1) and a split-inactivated vaccine bearing an H5 head domain and an H1 stem domain (cH5/1) (Table 1) [36]. The authors evaluated the protection against challenge with pandemic H1N1 virus in preclinical ferret studies following different sequential prime-boost combinations and immunization regimens. Collectively, these studies indicate that a sequential live-attenuated followed by split-inactivated virus vaccination approach confers superior protection against pandemic H1N1 infection. As speculated by the authors, these results can likely be attributed to the ability of LAIV to replicate in the upper respiratory tract, leading to an intracellular antigen expression, and superior priming of an adaptive cellular immune response.

**Table 1:** Advanced under development universal influenza vaccines

Vaccine approach	Company	Mechanism of protection	Study phase	References
Chimeric HA proteins	GlaxoSmithKline	• ADCC • Fusion inhibition	Clinical phase 1	[35, 36]
Computationally optimized broadly reactive antigens (COBRA)	Sanofi-Pasteur	Elicitation of HAI+ antibodies	Preclinical	[62, 64, 66]
NP, M1 and HA peptides (M-001)	BiondVax Pharmaceuticals Ltd	B cell- and T cell-mediated immune response	Clinical phase 2	[77–79]

List of universal influenza vaccine candidates discussed in this review and currently in an advanced stage of development

#### Stem-based immunogens

Analogously, minimized stem immunogens expressed in eukaryotic, as well as prokaryotic systems, efficiently elicit anti-stem antibodies. These antigens are resistant to thermal/chemical stress and thus make them a cost- and storage-affordable option. These mini-stem immunogens also elicit a heterosubtypic immune response, which protected mice from disease and death following a lethal challenge [28]. Similarly, Impagliazzo et al. generated stable mini-HA stem antigens based on the H1 subtype. The best candidate exhibited structural and binding properties with broadly neutralizing antibodies comparable to those of full-length HA, confirming its proper folding. Moreover, this immunogen completely protected mice in lethal heterologous and heterosubtypic challenge models and reduced fever after sublethal challenge in cynomolgus monkeys [37].

#### Mechanisms of neutralization elicited by stem-based approaches

The common denominator of these different approaches is skewing of the antibody response towards the HA stem. However, while targeting the conserved HA stem region is an attractive and feasible approach, a key issue is whether an antibody response directed towards HA stem epitopes would sufficiently protect against all circulating influenza strains. The ability of antibodies targeting conserved epitopes in the stem region to confer protection is still being evaluated. In fact, as demonstrated by Valkenburg et al., the mode of protection conferred by stem-directed antibodies is not directly related to lower viral replication or inflammation in the lung. Although these antibodies protect small animals from mortality, these vaccines failed to prevent infection or reduce lung viral titer [28]. In fact, a significant portion of the HA-stem antibodies induced by vaccination with mini-stem are non-neutralizing. Accordingly, a plethora of HA-stem directed monoclonal antibodies (mAbs) endowed with different recognition, neutralization and protection profiles have been described [38–40]. However, non-neutralizing antibodies may protect by recruiting other immune factors or cell types that mediate antibody-dependent complement cytotoxicity (ADCC) and alter viral membrane fusion during entry or otherwise interfere with the viral life cycle. In particular, interaction of the antibody constant region with different Fc-receptors (e.g. FcγRIII) could involve and activate other immune compartments, such as cell populations belonging to the innate immune response branch (NK and monocytes/macrophages). In this regard, it is difficult to evaluate ADCC-related mechanisms in animal models (i.e. mice) by using fully human mAbs. In order to overcome this limitation, in vitro models of ADCC evaluation (such as NK-based assays), substitution of the human antibody constant domains with murine constant domains or using transgenic mice expressing human Fc receptors, should be performed.

In order to rely more on ADCC-related mechanisms and overcome HA hypervariability, targeting other more conserved and exposed proteins such as the ectodomains of NA and M2 (M2e) proteins, could be a feasible and complementary approach instead of relying on a direct antibody-mediated neutralization mechanism [41]. In this regard, future vaccinal approaches should be evaluated on and capable of eliciting not only broad antibody specificities but also mechanisms which contribute to the global protection and neutralization of the infection.

#### Limits of stem-based approaches

However, with all its success, stem-based immunogens may have some limitations. Some vaccine-induced anti-stem antibodies can promote virus fusion and enhance influenza virus induced respiratory disease [42]. In addition, these antibodies may be self-reactive due to their polyreactive profile and the proximity of the HA stem region to the cell membrane [43]. In addition, these antibodies may have low affinity for the HA on the virion resulting in reduced association rates [43]. This phenomenon has already been demonstrated by the reactivity profile of certain mAbs recognizing the membrane-proximal external region (MPER) of the human immunodeficiency virus (HIV) gp41 envelope glycoprotein. In detail, antibodies belonging to the VH1–69 germline subfamily are well known to be particularly elicited by the HA stem region and more likely by a highly conserved α-helix, through a non-canonical CDRH1 and CDRH2 engagement [44, 45]. Moreover, in humans, VH1–69 germline encoding B-cells account for less than 2% of the total [46], while a VH1–69 bias it is well known to be associated with autoimmune perturbations [47–49], thus raising some doubts about the real efficacy and the safety of a possible HA stem region-based vaccinal approach. It is well known that the hydrophobicity of the binding domain, in particular at the level of the framework regions of polyreactive antibodies, such as those belonging to the VH1–69 subfamily, as well as long CDR sequences, can favour the binding to hydrophobic pockets on the envelope of different viruses. On the other hand, this peculiar characteristic can promote autoreactivity phenomena triggered by the cross-recognition of host cellular components, i.e. cellular membranes, leading to a self-antigen recognition [50, 51].

This property of polyreactivity will likely further reduce the number of B cells in the restricted repertoire that can bind the stem of HA. Furthermore, a protein or peptide-based universal vaccine approach, as supposed to be those relying on HA stem and similarly to current protein-based vaccines (e.g. hepatitis B virus and human papillomavirus vaccines), would require multiple close administrations, compared to a universal viral-based influenza vaccine (formulated as a LAIV or IIV), in order to properly boost the immune response and with consequent cost- and time-related issues [36].

Finally, it has been described that stem-based vaccinal approaches induce a very limited boosting of antibodies against the stem of HA [52]. One accredited hypothesis for this phenomenon is that pre-existing anti-stem antibodies could bind and mask stem epitopes and thus limit the boosting effect of anti-stem antibodies. In particular, these antibodies could recognize irrelevant stem epitopes and interfere with the elicitation of those directed against neutralizing epitopes in the stem [53, 54]. However, as suggested by Zarnitsyna and colleagues, the antibody titer can be altered by increasing the dose of stem-based antigens used in the vaccine and thus counteracting the effects of epitope masking and allow for the boosting of a stronger anti-stem antibody response [55]. These findings, along with an improved understanding of how the immune system responds to influenza infection and vaccination, has spurred great efforts on the stem-based cross-subtype (‘universal’) vaccine design.

### HA head-based approaches

Whether antibodies elicited against the stem region of HA are able to protect against influenza virus challenge in people is unclear. In contrast, antibodies directed against conserved or pivotal regions of the HA head, involved in crucial steps of the viral life-cycle are well known to protect from and neutralize influenza virus infection [56]. The canonical mechanism at the basis of viral neutralization and protection of these antibodies is their binding to epitopes overlapping the receptor binding site and thus blocking the early step of viral entry [57]. In particular, these antibodies are endowed with hemagglutination inhibition (HAI) activity [58]. More in detail, these antibodies, by interacting with the sialic acid binding region of HA, prevent the in vitro agglutination of red blood cells when incubated with influenza virus or HA. However, the sole role of these antibodies in conferring protection against circulating influenza virus strains and the possible contribution of other immune factors, such as those involved in expanding the breadth of recognition, are still under investigation [59].

In this regard, additional mechanisms could contribute to protection, and other mechanisms have been suggested to contribute to the neutralizing profile of head-directed antibodies. As an example, it has been demonstrated that these antibody specificities can prevent the propagation and the release of viral progeny independently from entry or genome replication inhibition mechanisms [60]. Furthermore, inhibition of the nucleus entry of the viral nucleoprotein (NP), has been demonstrated to be an additional mechanism of viral neutralization by head-directed antibodies [61].

#### Computationally optimized broadly reactive antigens (COBRAs)

As previously discussed, the head region is the most variable portion of HA and elicits antibodies that are often strain and/or subtype-specific [10, 58]. In order to overcome the high variability of influenza HA, in particular at the level of the head, our group described the generation of computationally optimized broadly reactive antigens (COBRAs) for the influenza HA [62].

The COBRA-based approach can be considered as a classic reverse vaccinology approach based on the multiple layering of consensus HA protein sequences, followed by the generation of a final consensus sequence that is able to recapitulate, in a unique protein, amino acid changes undergone by influenza virus during the past years to present [63]. Thanks to this approach, prototypes COBRA-based vaccines are able to elicit a humoral immune response that is able to protect against past, current and, theoretically, future circulating strains [64]. In fact, there are epitopes in the HA head domain that are not only conserved within a subtype, but conserved also among different subtypes (e.g. H1 and H3) [65]. Notwithstanding, HA-head-specific anti-H1/H3 antibodies could show a non-neutralizing profile in vitro, and they can protect against infection with H1N1 and H3N2 virus strains when administered before or after the challenge, as recently described by Lee and colleagues, suggesting an ADCC-mediated activity [53].

Interestingly, the COBRA strategy has been described for different influenza subtypes, demonstrating the flexibility of this approach in covering influenza viruses from multiple subtypes [62, 64, 66]. More in detail, immunization of mice with H1N1-based COBRA candidates, conferred broad HAI activity against a panel of 17 H1N1 viral strains. Moreover, challenge of immunized mice gave little or no detectable viral replication, as observed in those immunized with a matched licensed vaccine [64]. Similarly, previous studies describing the design and generation of H5N1-based COBRA, demonstrated that mice and ferrets, as well as nonhuman primates (Cynomolgus macaques) vaccinated with COBRA clade 2 HA H5N1 virus-like particles (VLPs) had broader HAI antibody titers recognizing different isolates representative of divergent subclades [62, 67]. Furthermore, all COBRA-vaccinated animals were protected following challenge with a clade 2.2 representative isolate. In particular, no virus was detected in the nasal and tracheal washes, and reduced lung inflammation and pathologic hallmarks were observed in COBRA-vaccinated macaques as compared to those immunized with a matched vaccine [62, 67].

#### Advantages and drawbacks of COBRA-based approaches

Similar to other universal vaccinal approaches, it remains to be determined whether vaccimation with a COBRA HA will confer protection against future circulating seasonal and/or pandemic strains. But, in this regard, analysis of serum from subjects primed in 2011/12 with conserved epitopes of HA, conferred an improved seroprotection and seroconversion against following circulating strains, such as those that caused the 2014/15 influenza epidemic and that were not known to circulate in 2011/12 [68]. Thus, in a similar way, COBRA could elicit an antibody response able to protect from future circulating strains.

Moreover, protection against different subtypes is unlikely to be achieved using a single immunogen but, more realistically, may require a combination of antigens. In fact, in contrast to stem-based approaches, the COBRA approach may maximize the breadth of antibody recognition against all strains of influenza in a subtype with a single immunogen or a ‘cocktail’ of COBRA HA representing different subtypes [64]. Furthermore, addition of an adjuvant, such as MF59, to current and future vaccines can contribute in expanding the antibody breadth of recognition [69].

As a further advantage, COBRA HA proteins can be displayed as full-length, trimerized molecules on the surface of a virus or VLP [70]. This allows for native folding of the HA glycoprotein and the full-display of it to the immune system with the possibility of eliciting and recalling antibody responses to conserved and neutralizing regions of HA and induce ADCC-related mechanisms.

Since people have pre-existing anti-influenza immunity, people vaccinated with the COBRA HA, which contains several epitopes representing past influenza strains, will be able to mount a recall of B memory cells resulting in a broadly-reactive humoral immune response [59]. This phenomenon has been observed in preclinical studies performed in ferrets [71]. Moreover, these candidate vaccines will be evaluated in upcoming clinical trials (Table 1). The versatility of this approach makes it applicable for the development of vaccines for other variable and exposed proteins of the virus (i.e. NA), as well as in the development of vaccines against other hypervariable viruses, such as HIV and hepatitis C virus (HCV) [72].

Finally, as a further important aspect in the rapid generation of influenza vaccines during epidemics and pandemics is that COBRA influenza viruses could be used in the generation of LAIV or IIV vaccines (less expensive if compared to a unique or multiple recombinant protein-based immunogens) with the further possibility of implementing their high-yield production by using RNA segments (i.e. internal genes of high-yield replicating strains) [73]. In fact, in the case of a LAIV, infection of humans with influenza virus induces immune responses of greater quality, quantity and longevity compared to IIV [74]. Moreover, as recently demonstrated by our group in a preclinical model, differently from a VLP-based influenza vaccination, influenza infection with H1N1 or H3N2 strains elicits a lambda light chain-biased antibody response [75]. Evaluating the presence of this phenomenon in the context of a COBRA-based virus infection could help in the understanding of the immune response against these antigens and help in the development of a LAIV-based COBRA vaccine.

As a further application, the COBRA-based approach could have an additional application in the field of drug discovery. In vivo experiments showed that COBRA-based vaccines are able to elicit a cross-reactive and cross-protective humoral immune response. In this regard, it can be hypothezed that antibodies, as well as other molecules, that are able to bind COBRA, can be endowed of cross-reactive properties against different strains of HA which are recapitulated in the COBRA. In this case, such antigens could also be used for screening and selection of novel drugs endowed with cross-protective and cross-neutralizing properties. As an example, antibody or small molecules libraries could be screened and selected on COBRA-based antigens [65].

#### Other HA head-based approaches

In addition to the COBRA-based approach, there are other candidate vaccines focused on the HA head. In this regard, Song et al. described the generation of a fusion protein composed of the globular HA head domains (HA1–2, spanning amino acids 62–284) from H7N9 and the *Salmonella typhimurium* flagellin (fliC) expressed in *Escherichia coli (E. coli)* [76]. In particular, the authors chose fliC as being a potent Toll-like receptor-5 (TLR5) ligand in order to trigger an innate immune response with a consequent induction of cytokine production and dendritic cell activation eventually leading to higher titers of antigen-specific IgG. After having assessed the correct folding of the fusion protein, the authors found that it was able to elicit a significant and robust HA1–2-specific serum IgG titers, lasting for at least 3 months in the vaccinated animals, as well as an HA1–2-specific IgG1 and IgG2a response detectable 12 days after the third immunization. Finally, the HA1–2-fliC fusion protein was also found to be capable of triggering the production of HAI antibodies [76].

As an additional noteworthy vaccinal approach, the epitope-based Multimeric-001 (M-001) candidate vaccine is currently being evaluated in clinical trials (Table 1). This vaccine, firstly described by Ben-Yedidia et al. and further developed by *BiondVax Pharmaceuticals Ltd.*, is composed of B- and T-cell epitopes comprising nine conserved epitopes from the HA (including the globular head), NP and M1 proteins, derived from influenza A and B strains [77, 78]. As previously seen with COBRA, in which all the specifities are recapitulated in a unique antigen, in order to overcome the low immunogenicity and the high costs of M-001 peptides, the epitopes are combined in triplicate into a single recombinant protein expressed in *E. coli*. M-001 has been tested in both preclinical and clinical studies, conferring protection in mice against infection with different influenza strains and being safe and inducing both B- and T-cell specific immune responses, respectively [78].

However, M-001 per se is not able to elicit HAI antibodies which can be induced only when the administration of M-001 is followed by a boosting with seasonal or pandemic strain specific vaccines [79].

### Anti-idiotypic antibodies

In addition to the main head- and stem-based approaches above discussed, there are other vaccine candidates which deserve consideration and are in an early stage of development. Among them, vaccines based on the concept of anti-idiotypic antibodies represent an interesting and promising approach for the prophylaxis of influenza infection as well as other pathogen- and non pathogen-related diseases [80].

**Fig. 1:**
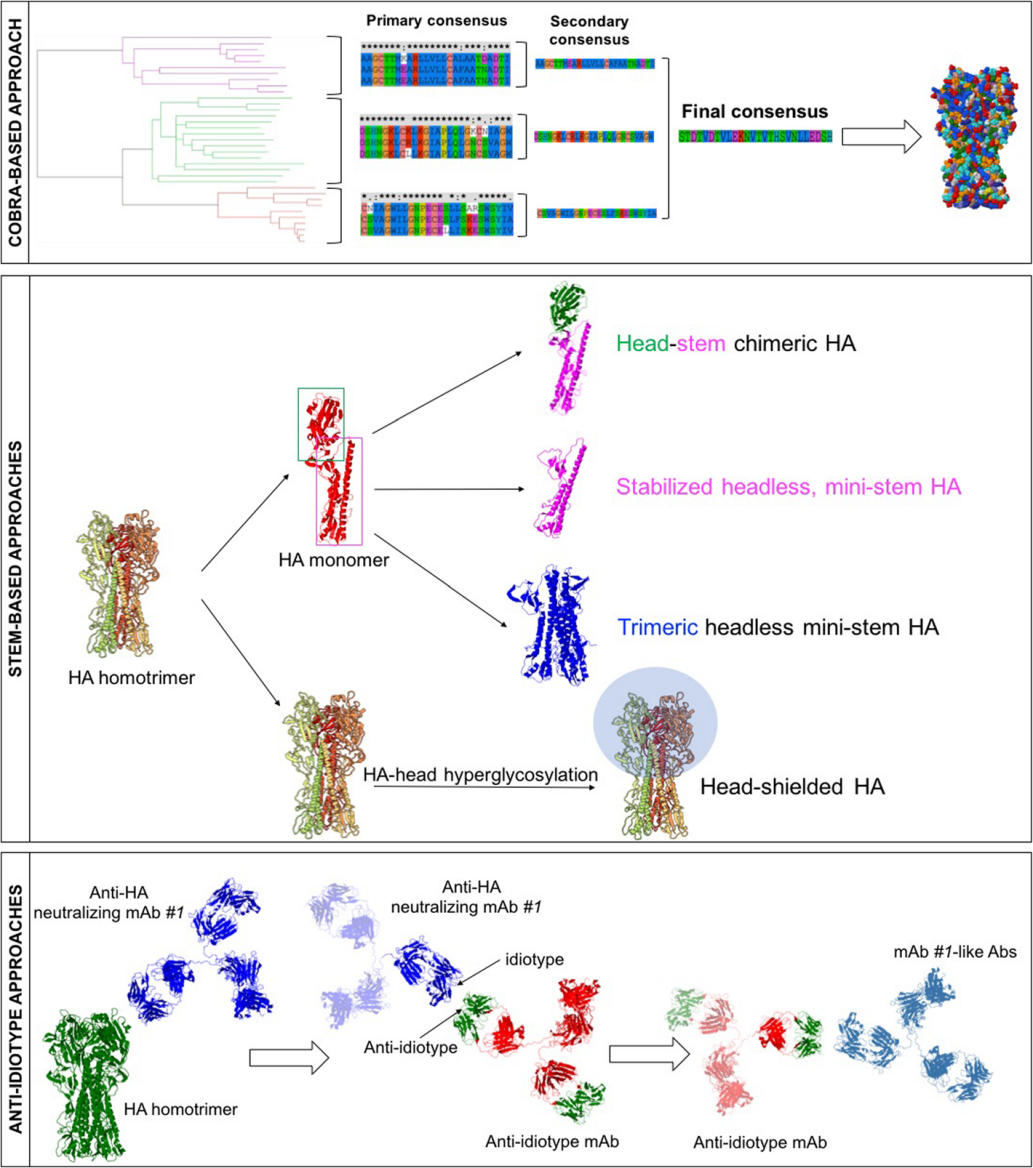
Representation of ‘universal’ vaccine approaches under development. Top panel: schematic representation of COBRA-based approach. A phylogenetic tree is inferred based on hemagglutinin (HA) amino acid sequences. Primary and secondary consensus sequences are thus generated. Finally, the secondary consensus sequences are then aligned and the resulting consensus, designated COBRA, is generated. Central panel: schematic representation of approaches aimed at eliciting/boosting an antibody response against the HA stem region. These strategies rely on the chimerization of the HA molecule in order to direct the antibody response towards the stem region or on the masking of the head region (i.e. through the hyperglycosylation of the HA head). Bottom panel: schematic representation of anti-idiotype based approaches. As an example, a monoclonal antibody (mAb #1) recognizing a conserved and protective/neutralizing epitope of the HA molecule is used as a footprint antigen to elicit antibodies recognizing the idiotype of the original antibody (mAb #1). The best candidate anti-idiotype antibody able to elicit antibodies having similar binding and neutralizing characteristics of mAb #1 is then selected as immunizing antigen to develop epitope-based vaccine approaches

This approach could be considered another branch of the reverse vaccinology (for this reason also called reverse vaccinology 2.0) and is based on the generation of anti-idiotypic antibodies by using broadly neutralizing mAbs as a footprint. In brief, a mAb recognizing a conserved and protective/neutralizing epitope of the HA molecule is used as the immunizing antigen, in a different species animal model, to elicit antibodies recognizing the idiotype of the original antibody. Anti-idiotype antibodies are then selected based on their binding and neutralizing properties. Ideally, generated anti-idiotypic mAbs should elicit a humoral immune response characterized by the presence of antibodies with similar binding and neutralizing properties to the mAb used to generate them. These antibodies will be then used to develop epitope-based vaccine approaches (Fig. 1).

This approach has been proposed for different pathogens like HIV, fungi and also influenza virus [55, 81–84]. In particular, for influenza virus, Li and colleagues described the generation of an anti-idiotypic antibody for the avian H9 HA subtype by immunizing BALB/c mice with purified chicken anti-H9 IgG and generated specific B-cell hybridomas [84]. After screening of the hybridomas against both chicken and rabbit anti-H9 IgG, the authors identified a mAb (named mAb2) that was able to inhibit the binding of hemagglutinin to anti-H9 IgG and to induce chickens to generate HAI antibodies, indicating the specific binding of this mAb to the idiotype of anti-H9 IgG.

### Other influenza virus targets

Protection elicited by the current seasonal influenza vaccine is predominantly antibody-mediated [85–87]. A key issue for future under-development vaccines is the capacity to elicit a more complete immune response, in particular those involving other branches of the immune system, such as the innate and T cell arms [88]. As an example, approaches focused on the elicitation of immune responses directed against more conserved influenza proteins such as M2 or nucleoprotein (NP) are in development. However, these vaccines appear to only modulate disease severity and fail to prevent morbidity and mortality following a high-dose influenza virus challenge [89–92]. Similar to the epitope-based M-001 vaccine, other approaches aim at combining conserved viral peptides in order to elicit an heterosubtypic immune response. In this regard, Guo et al., constructed two recombinant protein vaccines by respectively linking highly conserved sequences from two M2e domains and one domain corresponding to the FP domain of HA of H5N1 and H7N9 influenza viruses in different orders [93]. The authors demonstrated that these *E. coli* derived immunogens induced high-titer M2e-FP-specific antibodies in immunized mice. Moreover, immunization with M2e-FP prevented lethal challenge of an heterologous H1N1 influenza virus, with significantly reduced viral titers and alleviated pathological changes in the lungs, as well as increased body weight and complete survivals, in the challenged mice. However, in this paper, the authors analyzed only the extent of the humoral immune response elicited by the immunizing antigens they used. As discussed by the authors, antibody- (e.g. ADCC) as well as T cell-dependent responses should be investigated in order to understand the immune-mediated mechanisms at the basis of the protection conferred by this kind of immunogens. In this regards, future influenza vaccines should be evaluated on and capable of recruiting the other compartments of the immune response, in particular the T-cell mediated immune response, that can synergize with that conferred by B cells.

## Conclusions

In the last decade, there has been a great effort to develop the so-called ‘universal’ influenza vaccine. Different approaches have been developed to reach this goal and curb the outbreak of possible influenza epidemics and pandemics. The more promising candidates have recently entered or are going to enter in the clinical phase iter (Table 1).

As a final remark, new approaches should be focused also on the activation of the naïve B cells compartment, and the generation of antibodies directed against neutralizing epitopes, particularly in those populations undergoing immunosenescence, such as in the elderly [94]. Any new broadly-reactive or universal influenza vaccine will need to be effective in all populations including children, the elderly, pregnant women and immunocompromised people. Moreover, prevalence and breadth of the antibody cross-reactivity of the general human population varies and largely depends on the individual history of exposure to influenza viruses [95]. In fact, it has been observed that the first HA subtype to which a person is exposed leaves an immunological imprint that will substantially affect the antibody cross-reactivity that this person develops, the so-called ‘original antigenic sin’. This phenomenon should be taken into account in order to develop a ‘universal’ influenza vaccine and shed light on the possibility of developing personalized or group-related immunogens.

The goal of developing broadly-reactive or universal influenza vaccines with the ability to protect against co-circulating strains is within reach. Finding a strategy that could overcome this enormous variability in viral proteins and making a vaccine effective is challenging. Any approach will need to take into account the diversity of influenza virus proteins and may need the use of multiple vaccines conferring a homosubtypic protection (e.g. against H1 or H3 in the case of influenza virus) and a heterosubtypic protection (against either H1 and H3) as a ‘cocktail’ formulation, rather than a single immunogen [96]. Prophylactic, as well as therapeutic approaches, aimed at targeting a single region or epitope of the antigen can be more easily circumvented by the pathogen and thus lead to a compromised effectiveness. On the other hand, approaches directed towards multiple targets are difficultly escaped by the pathogen. This is evident when considering the therapeutic approaches against hypervariable pathogens like HIV and HCV [97]. Current available treatments to these viruses are directed against multiple targets (e.g. viral proteins, such as the polymerase and the protease), while first generation antiviral drugs against these viruses were focused only on one target [98]. Similarly, drugs against influenza virus target the NA and M2 proteins. However, there are basically two kind of drugs directed against these targets: the adamantanes and NA inhibitors, which are a few when compared to those available for HIV and HCV. This low spectrum of available drugs for influenza virus is reflected in the easy occurrence of escape variants when administered.

As for therapeutic approaches, the development of prophylactic approaches against hypervariable pathogens should be focused on multiple targets/epitopes. However, in the case of vaccines, the spectrum of possible targets is reduced when considering protection as the final goal. Viral surface HA and NA antigens are the main immune targets of most influenza vaccines.

However, current available influenza vaccines are mainly HA-based. In fact, while HA content is determined and standardized, the content of NA is not quantified during the manufacturing process of IIV. In fact, like HA, NA plays a key antigenic role in the host immune response and it has been demonstrated that serum NA-inhibiting antibody titer positively correlates with vaccine effectiveness [99, 100].

Finally, multiple B-cell epitopes, at the level of the HA head region (including the receptor binding site), as well as of the stem region, can neutralize the virus and confer protection. Thus, an influenza vaccine eliciting a higher spectrum of protective antibodies could be more effective and hamper the occurrence of possible drift variants, compared to those based on a single region/epitope.

The next few years will be an exciting time as vaccine based on stem and globular head of the HA move from pre-clinical to clinical studies. The most promising vaccines under development will enter in the clinical evaluation in the next 5 years. These clinical studies could represent the final testbed of their effectiveness by demonstrating their possible ability to protect people against co-circulating influenza strains from multiple subtypes compared to currently available commercial vaccines. In particular, they will provide a more complete understanding of their effect in a pre-immune context in humans, as well as the ability to understand the biomarkers and the molecular signatures linked to protection in humans.

## Abbreviations

Ab: Antibody
ADCC: Antibody-dependent complement cytotoxicity
COBRA: Computationally optimized broadly reactive antigens
FP: Fusion peptide
GISRS: Global Influenza Surveillance and Response System
HA: Hemagglutinin
HAI: Hemagglutination inhibition
HCV: Hepatitis C virus
HIV: Human immunodeficiency virus
IIV: Inactivated influenza vaccine
LAIV: Live attenuated influenza vaccine
M2: matrix protein 2
M2e: M2 ectodomain
mAb: monoclonal antibody
MPER: Membrane-proximal external region
NA: Neuraminidase
NP: Nucleoprotein
RBS: Receptor binding site
TLR5: Toll-like receptor-5
VLP: Virus-like particle
WHO: World Health Organization.
